# Minor Hospital War News

**Published:** 1914-08-29

**Authors:** 


### Minor Hospital War News.

SEA COPSE HILL, Whippingham, Isle of Wight, a freehold residence, with grounds of nine acres, has been presented by Mrs. Frederick J. Kirchner as a free gift to the Royal Waterloo Hospital for Children and Women, to be used as a convalescent home.

SUBSCRIPTIONS are asked for the Duchess of Westminster's War Hospital, 82 Harley Street, W., which is being established with the approval of the War Office and the Red Cross Society, under the charge of Major H. E. M. Douglas, R.A.M.C., V.C. It will cost £6,000 to equip, and £1,400 per month for the maintenance of the 200 beds to be contained; £1,743 has already been received, and £600 per month guaranteed.

THE Committee of Management of Queen Charlotte's Lying-in Hospital have arranged, as on former similar occasions, to admit the wives of sailors and soldiers on active service without the usual subscriber's letter of recommendation, or they may, if they prefer it, be attended by the hospital midwives in their own homes, provided they live within the hospital out-patient districts.

A HOSPITAL for sick and wounded soldiers has been decided upon at Melton Mowbray. Mr. R. Dalgleish, D.L., J.P., Chairman of the Territorial Association of Leicestershire, presided at a recent meeting. It would consist of thirty beds, and North Lodge has been placed at their disposal by Mrs. Powell, rent free, during the period of the war, while Mrs. Stevenson has promised to accommodate the nurses and lend the use of her kitchen if necessary. Dr. Tibbles stated that the medical men of the town would undertake the medical treatment of the soldiers and attend necessitous dependants of all Reservists and Territorials called up. They would also organise classes to help the St. John Ambulance and other Associations.

THE Middlesex Hospital has started a first-aid class for ladies only, under the direction of Mr. Somerville Hastings. The class is held daily at 5.30, and no fee is charged.

THE QUEEN having accepted the presidency of Queen Mary's Royal Naval Hospital, Southend-on-Sea, has sanctioned an appeal to the Empire in her name for funds to support the hospital.

SIR ROBERT LUCAS-TOOTH has given £10,000 to Lady Dudley towards forming the fund for the Australian voluntary hospital for war service, organised by her and accepted by the War Office. Sir Robert Lucas-Tooth has also promised to act as chairman of the committee.

### PERSONALIA.

DR. JAS. KEMP, M.R.C.S., L.R.C.P., an old "Bart.'s" man, has volunteered for active service, and has been called up.

DR. LIONEL WOODS, L.R.C.P.I., L.R.C.S.I., assistant consulting surgeon to Noble's (Isle of Man) Hospital, has volunteered for active service.

MR. R. A. OWTHWAITE, whose work as an ex-hospital officer was referred to last week, was formerly, it should have been stated, secretary of the City of London Lying-in Hospital.

DR. GEORGE FRENCH STEBBING, M.B., B.S., L.R.C.P. (Lond.), M.R.C.S. (Eng.), assistant medical officer at Lambeth Infirmary, has joined H.M.S. *Leviathan* as surgeon. He was formerly at Guy's Hospital.

